# Culture Conditions Shape the Phenotype and Quality of *Talaromyces sayulitensis* HC1 Conidia

**DOI:** 10.3390/jof12080628

**Published:** 2026-08-21

**Authors:** Ivonne Gutiérrez-Rojas, Paola Ruiz-García, Julián D. Camargo-Pacanchique, María X. Rodríguez-Bocanegra, Alba Avila, Homero F. Pastrana, Ibonne Aydee García Romero, Zulma Suárez-Moreno, Nubia Moreno-Sarmiento

**Affiliations:** 1 Grupo de Biotecnología Ambiental e Industrial (GBAI), Departamento de Microbiología, Facultad de Ciencias, Pontificia Universidad Javeriana, Bogotá 110231, Colombia; ruiz.gina@javeriana.edu.co; 2 Centro de Microelectrónica (CMUA), Departamento de Ingeniería Eléctrica y Electrónica, Universidad de los Andes, Bogotá 111711, Colombia; a-avila@uniandes.edu.co (A.A.); hf.pastrana122@uniandes.edu.co (H.F.P.); 3 Unidad de Investigaciones Agropecuarias–UNIDIA, Departamento de Microbiología, Facultad de Ciencias, Pontificia Universidad Javeriana, Bogotá 110231, Colombia; julian.camargo@javeriana.edu.co (J.D.C.-P.); mxrodriguez@javeriana.edu.co (M.X.R.-B.); 4 Grupo de Bioprocesos y Bioprospección, Instituto de Biotecnología, Universidad Nacional de Colombia, Bogotá 111321, Colombia; iagarciar@unal.edu.co (I.A.G.R.); ncmorenos@unal.edu.co (N.M.-S.); 5 Departamento de Investigación y Desarrollo, Empresa Colombiana de Productos Veterinarios, VECOL S.A., Bogotá 110931, Colombia; zulma.suarez@vecol.com.co

**Keywords:** filamentous fungi, fungal propagules, solid-state fermentation, submerged fermentation, conidial quality, germination, thermotolerance, fungal inoculants

## Abstract

This study evaluated how culture system and culture medium composition influence the phenotype and functional quality of *Talaromyces sayulitensis* HC1 conidia. Conidia were produced by solid-state fermentation (SSF) and submerged fermentation (SmF) using two culture medium compositions differing in sucrose and ammonium phosphate concentrations and, consequently, in C:N ratio. Morphological, ultrastructural, physicochemical and functional attributes were analyzed together with the expression of four selected conidiation-related genes (*brlA*, *abaA*, *fluG* and *rodA*). Conidia produced under SSF were significantly smaller and displayed a more cylindrical morphology and accumulated significantly more melanin than those produced under SmF, whereas SmF-derived conidia exhibited significantly higher relative ergosterol content. Outer cell wall thickness was significantly influenced by both culture system and culture medium composition. Across both culture systems, the medium containing a lower sucrose concentration and a higher ammonium phosphate concentration (C:N ratio 10:1) resulted in significantly higher germination but significantly lower thermotolerance than the medium containing a higher sucrose concentration and a lower ammonium phosphate concentration (C:N ratio 50:1). The medium was also associated with significantly greater melanization. Expression of *brlA*, *abaA*, *fluG* and *rodA* was generally higher under SSF, although the magnitude of the response varied among genes and culture medium compositions. The integrated analysis revealed that germination and thermotolerance were not necessarily coupled and that culture system and culture medium composition influenced different dimensions of conidial quality. The main innovation of this study is the integration of structural, physicochemical, functional and selected molecular indicators to identify distinct conidial quality profiles relevant to application-oriented production of fungal inoculants.

## 1. Introduction

*Talaromyces sayulitensis* HC1 (formerly identified as *Penicillium* sp. HC1) is a filamentous fungus isolated from rice-cultivated soils that exhibits high cellulolytic and xylanolytic activity [1,2]. Owing to its lignocellulose-degrading capacity, this microorganism has been investigated for the biodegradation of agricultural residues such as rice straw [1] and as a component of a tripartite bioinoculant consortium with *Bacillus* spp. for composting processes [3]. To support its future development as a fungal bioinoculant, the production of high-quality conidia is essential. Unlike vegetative hyphae, which are responsible for fungal growth and substrate colonization, conidia are specialized asexual spores adapted for dispersal, survival and the initiation of new fungal colonies. Compared with vegetative hyphae, conidia possess structural and physiological traits that enhance their tolerance to environmental stresses, including low water content, specialized cell wall architecture and composition, accumulation of protective carbohydrates and pigments, and increased resistance to thermal, oxidative and UV stress [4]. These characteristics make conidia suitable propagules for the formulation, storage and commercialization of fungal bioproducts, where conidial stability and stress tolerance are critical quality attributes influencing product shelf life and field performance.

Solid-state fermentation (SSF) has traditionally been regarded as a suitable platform for the production of fungal conidia because it provides environmental conditions that more closely resemble the natural habitats of filamentous fungi, including growth on solid surfaces and low water availability [5,6]. Under these conditions, aerial development and conidiation are often favored, and numerous fungal species produce higher conidial yields than under submerged fermentation (SmF), although these responses remain strongly species- and condition-dependent [5,7].

Despite these biological advantages, the industrial implementation of SSF remains challenging because of difficulties in process monitoring and control, the formation of temperature, moisture and oxygen gradients within the substrate bed, and the complexity of biomass recovery and downstream processing. In contrast, SmF offers important operational advantages, including improved process control, greater reproducibility, easier monitoring of culture conditions, and more straightforward scale-up in industrial bioreactors [8,9]. However, these advantages can be fully realized only if conidia produced under submerged conditions possess the quality attributes required for their intended industrial application. Previous studies on *Talaromyces sayulitensis* HC1 demonstrated that culture system and culture medium composition influence not only conidial production but also conidial tolerance to environmental stresses [10]. These findings are consistent with increasing evidence showing that culture conditions shape multiple attributes associated with conidial quality in filamentous fungi [11,12,13]. Consequently, understanding how culture conditions influence conidial quality has become increasingly important for the development of fungal bioproducts.

This quality is determined by multiple structural, physicochemical and physiological characteristics associated with stress tolerance, environmental persistence and biological performance. Among these, surface properties—including hydrophobicity, electrostatic charge and adhesion capacity—are strongly influenced by the composition and organization of the outer cell wall, particularly hydrophobin-based rodlet structures, which play a key role in defining the physicochemical behavior of conidia [14,15]. Additional structural and biochemical components also contribute to conidial quality. Melanin deposition protects conidia against ultraviolet radiation, oxidative stress and desiccation, whereas membrane sterol composition, particularly ergosterol, is essential for membrane integrity, conidial development and adaptation to adverse environmental conditions [16,17]. Likewise, compatible solutes such as trehalose and mannitol, accumulated during conidial maturation, are major determinants of heat tolerance and have been positively associated with increased conidial survival under thermal stress [18]. Together, these characteristics determine the stability, storage performance and biotechnological potential of fungal conidia, highlighting the importance of understanding the mechanisms by which culture conditions shape conidial quality.

The development and maturation of fungal conidia are governed by hierarchical regulatory networks that integrate environmental and nutritional signals. In the extensively studied model *Aspergillus nidulans*, *fluG* acts as an upstream developmental activator involved in initiating the transition from vegetative growth to conidiation, whereas *brlA* and *abaA* form part of the central regulatory pathway that sequentially controls conidiophore development, phialide differentiation and conidial formation [19,20]. Beyond regulating conidiogenesis itself, this developmental hierarchy controls genes underlying conidial structural and functional characteristics. For example, BrlA regulates developmental genes including *abaA* and *rodA*, the latter encoding the hydrophobin responsible for the rodlet layer that contributes to conidial surface hydrophobicity, whereas AbaA activates downstream regulators involved in conidial maturation [19,20]. Moreover, fungal gene expression is strongly influenced by culture conditions and nutrient availability. For example, *Aspergillus niger* grown under solid and submerged fermentation exhibits distinct transcriptomic responses, including differences in the expression of transcription factors and metabolic genes [21]. Similarly, nutrient limitation during submerged fermentation of *Antrodia cinnamomea* promoted sporulation and altered the expression of developmental regulators associated with the *brlA–abaA–wetA* regulatory pathway [22]. Together, these findings support the concept that culture conditions may influence not only conidial production but also the developmental programs that ultimately shape conidial quality.

Despite these advances, current knowledge has largely followed two complementary research directions. The molecular regulation of conidiogenesis has been elucidated primarily in the model fungus *Aspergillus nidulans*, whereas the effects of culture conditions on conidial quality and subsequent biological performance have been investigated predominantly in entomopathogenic fungi. As a result, considerably less information is available for fungal species with biotechnological applications outside these well-studied groups. In particular, little is known about how culture systems and culture medium composition influence both the molecular regulation of conidiogenesis and the physicochemical and functional determinants of conidial quality in *Talaromyces* species. This gap is especially relevant for *Talaromyces sayulitensis* HC1, whose successful development as a fungal bioinoculant requires a better understanding of the mechanisms by which culture conditions shape conidial phenotype and quality.

Therefore, this study aimed to evaluate the effects of culture system and culture medium composition on the phenotype and functional quality of *Talaromyces sayulitensis* HC1 conidia. To this end, conidia produced under solid-state and submerged fermentation using two culture medium compositions differing in sucrose and ammonium phosphate concentrations and, consequently, in carbon-to-nitrogen ratio were characterized through morphological, ultrastructural, physicochemical and functional analyses. Morphological and ultrastructural characterization included scanning electron microscopy (SEM), atomic force microscopy (AFM) and transmission electron microscopy (TEM). Physicochemical characterization comprised the evaluation of electrostatic properties, melanin and ergosterol contents. Functional quality was assessed based on germination capacity and tolerance to thermal stress. In addition, the expression of selected genes involved in conidiation was evaluated as a complementary approach to investigate the molecular response to the different culture conditions.

## 2. Materials and Methods

### 2.1. Experimental Design and Conidia Production

A 2^2^ factorial experimental design was used to evaluate the effects of culture system and culture medium composition on the quality of *Talaromyces sayulitensis* HC1 conidia. The evaluated factors were culture system, including solid-state fermentation (SSF) and submerged fermentation (SmF), and culture medium composition, represented by two compositions (Table 1). These culture medium compositions differed in sucrose concentration, ammonium phosphate concentration and, consequently, carbon-to-nitrogen (C:N) ratio. The selection of these compositions was guided by previous findings obtained with the same fungal isolate (formerly identified as *Penicillium* sp. HC1), which demonstrated that culture medium composition and C:N ratio strongly influence conidial production and quality [10]. Therefore, the objective of this study was not to isolate the individual effects of sucrose concentration, ammonium phosphate concentration or C:N ratio, but rather to compare conidial quality between the two culture medium compositions evaluated. Conidia produced under each culture condition were subsequently characterized in terms of their structural, physicochemical and functional attributes, and the expression of selected conidiation-related genes was evaluated as a complementary approach.

All culture media contained the same mineral salt base (per liter: 0.5 g MgSO_4_, 0.5 g KCl, 1.0 g K_2_HPO_4_, 0.5 g CaCO_3_, and 100 μL of a trace element solution). The trace element solution contained (g L^−1^): FeSO_4_·7H_2_O, 2.0; CaCl_2_, 2.0; CoCl_2_·6H_2_O, 0.2; CuCl_2_·2H_2_O, 0.01; NiCl_2_·6H_2_O, 0.2; MnCl_2_·4H_2_O, 0.03; ZnSO_4_·7H_2_O, 0.1; H_3_BO_3_, 0.3; and Na_2_MoO_4_·2H_2_O, 0.03, prepared in 0.1 N HCl. Sucrose and (NH_4_)_2_HPO_4_ concentrations were adjusted according to the experimental condition (Table 1). The pH of all media was adjusted to 6.5 prior to sterilization. For submerged fermentation, 250 mL Erlenmeyer flasks containing 100 mL of medium were inoculated with 10 mL of a conidial suspension adjusted to 1 × 10^8^ conidia mL^−1^ by counting with an improved Neubauer hemocytometer and incubated at 28 °C and 150 rpm in an Innova^®^ 42 incubator shaker (New Brunswick Scientific, Edison, NJ, USA). For solid-state fermentation, culture media were supplemented with agar (15 g L^−1^) and inoculated at the center of each Petri dish with 50 μL of the same conidial suspension. Petri dishes were incubated at 28 °C in a FOC 225E refrigerated incubator (VELP Scientifica, Usmate Velate (MB), Italy).

Cultures were incubated at 28 °C, and conidia from both culture systems were harvested after eight days of incubation. Each experimental condition was performed using five independent biological replicates. This incubation period was selected because previous experimental observations with *Talaromyces sayulitensis* HC1 indicated that conidia produced under SSF reached physiological maturity after eight days; therefore, the same incubation period was adopted for SmF to enable a standardized comparison between the two culture systems. Conidia produced under SSF were harvested by gently scraping the colony surface with 0.85% (*w*/*v*) saline solution containing 0.1% (*v*/*v*) Tween 80. The resulting suspension was passed through a sterile cotton plug packed inside a syringe to remove hyphal fragments, centrifuged, and washed twice with 0.85% (*w*/*v*) saline solution. Conidia produced under SmF were recovered by centrifugation and washed twice with 0.85% (*w*/*v*) saline solution to remove residual culture medium. Additional sample preparation, where applicable, is described in the corresponding sections.

### 2.2. Characterization of Conidial Phenotype and Quality

Conidial phenotype and quality were characterized through structural, physicochemical, functional and molecular analyses. Morphological and ultrastructural characterization included scanning electron microscopy (SEM), atomic force microscopy (AFM) and transmission electron microscopy (TEM) to evaluate conidial surface morphology, topography, mechanical properties and cell wall ultrastructure. Physicochemical characterization comprised zeta potential determination and quantification of melanin and ergosterol content, which were used as indicators of surface electrostatic properties, stress-protective components and membrane composition, respectively. Functional quality was assessed through germination and thermotolerance assays. Detailed methodologies for each analysis are described in the following sections.

#### 2.2.1. Structural Characterization

##### Scanning Electron Microscopy (SEM)

Scanning electron microscopy (SEM) was used to evaluate the surface morphology of conidia produced under different culture conditions. Sample preparation was performed following a previously described protocol for fungal samples, with modifications [23]. Conidia were harvested after 8 days of incubation, washed twice with phosphate-buffered saline (PBS, 0.1 M, pH 7.0) and fixed in 2.5% (*v*/*v*) glutaraldehyde prepared in the same buffer for 48 h at 4 °C. After fixation, samples were washed three times with PBS and dehydrated through a graded ethanol series (70, 80 and 90%, 20 min each, two washes per concentration), followed by incubation in absolute ethanol (100%) for 30 min. The samples were dried using a critical point dryer (Samdri 795, Tousimis, Rockville, MD, USA) and sputter-coated with a 5 nm gold layer using a Desk IV sputter coater (Denton Vacuum, Moorestown, NJ, USA). Micrographs were acquired using a JSM-6490LV scanning electron microscope (JEOL Ltd., Akishima, Tokyo, Japan) operated at accelerating voltages of 10–20 kV, using a 25 µm aperture and a working distance of 3.5 mm. Images were recorded at a resolution of 3072 × 2304 pixels. Morphometric measurements, including conidial area and circularity index, were performed on approximately 80 conidia per treatment from SEM micrographs using ImageJ software version 1.53g (National Institutes of Health, Bethesda, MD, USA).

##### Atomic Force Microscopy (AFM)

Atomic force microscopy (AFM) was used to evaluate the surface topography and mechanical properties of conidia produced under different culture conditions. Conidial suspensions were deposited onto poly-L-lysine-coated surfaces and allowed to adhere prior to analysis. Topographic images were acquired using an MFP-3D BIO atomic force microscope (Asylum Research, Santa Barbara, CA, USA) operated in tapping mode with silicon AFM cantilevers (AC240TS, Olympus Corporation, Tokyo, Japan) with a nominal spring constant of 2 N m^−1^ and a tip radius of approximately 7 nm. Images were collected at 256 lines and a scan rate of 0.5 Hz.

The elastic modulus (Young’s modulus, *E*) was determined using the force-volume mode with a maximum applied force of 5 nN and an effective indentation depth of 10%. For each biological replicate, five conidia were analyzed, and three measurements were performed at the same location on each conidium. Young’s modulus was calculated using the Sneddon model, which describes the indenter as an ideal cone, according to Equation (1) [24,25,26].

(1) $$F=\frac{2}{\pi }tan(\alpha )\frac{E}{1-\nu^{2}}\delta^{2}$$

where *F* is the applied force, *E* is Young’s modulus, *ν*^2^ is the square of Poisson’s ratio (*ν* was assumed to be 0.5 for soft biological materials), *δ*^2^ is the square of the indentation depth, and *α* is the half-angle of the cone.

##### Transmission Electron Microscopy (TEM)

Transmission electron microscopy was performed using a JEOL 1400 Plus transmission electron microscope (JEOL, Tokyo, Japan) to evaluate the ultrastructure of conidia produced under different culture conditions. Conidia harvested after 8 days of incubation were washed twice with phosphate-buffered saline (PBS) and fixed in 3.4% (*v*/*v*) glutaraldehyde prepared in PBS for 48 h. Samples were subsequently post-fixed in osmium tetroxide for 2 h at room temperature and washed three times with PBS. Dehydration was performed through a graded ethanol series (70, 80 and 90%, 20 min each, two washes per concentration), followed by incubation in absolute ethanol (100%) for 30 min. Samples were then infiltrated and embedded in Spurr^®^ resin (Sigma-Aldrich, St. Louis, MO, USA) and ultrathin sections were mounted on 200-mesh copper grids.

#### 2.2.2. Physicochemical Characterization

##### Zeta Potential Determination

Zeta potential was determined as an indicator of the surface electrostatic properties of conidia produced under different culture conditions. Conidial suspensions obtained from 8-day cultures were adjusted to 5 × 10^6^ conidia mL^−1^ in 0.1 M phosphate buffer (pH 7.0). Measurements were performed using a Zetasizer Nano ZS instrument (Malvern Panalytical Ltd., Malvern, Worcestershire, UK). Aliquots of conidial suspension (>2 mL) were transferred to a quartz measurement cell, and the zeta potential was determined, with each biological replicate measured in technical triplicate. The mean zeta potential value was subsequently calculated for each treatment.

##### Relative Estimation of Melanin Content

Relative melanin content was estimated as an indicator of conidial protection against environmental stress. Melanin extraction was performed according to Selvakumar et al. (2018) [27]. Briefly, freshly harvested conidia were washed and resuspended in 0.85% (*w*/*v*) saline solution to a final concentration of 3 × 10^8^ conidia mL^−1^. Conidial suspensions were treated with 0.1 M NaOH and autoclaved at 121 °C and 17 psi for 15 min. The resulting suspension was centrifuged at 5000× *g* for 15 min to remove coarse debris. The supernatant was acidified with concentrated HCl and stirred at 120 rpm for 10 min to promote melanin precipitation. The melanin precipitate was recovered by centrifugation at 5000× *g* for 15 min and washed with distilled water to remove residual acid. After air drying, the pigment was resuspended in borate buffer (pH 8.0).

The spectrophotometric profile of the extracted pigment was recorded between 200 and 500 nm and compared with that of a commercial melanin standard (Sigma-Aldrich M8631) to assess spectral similarity (Figure S1). Absorbance was measured at 218 nm, corresponding to the maximum absorption peak of the commercial standard, and a calibration curve prepared with commercial melanin (Sigma-Aldrich M8631) was used to estimate the relative melanin content of the samples. The assay was intended for comparative analysis among treatments and was not validated for absolute quantification of melanin in *Talaromyces sayulitensis* HC1 conidia.

##### Relative Estimation of Ergosterol Content

A relative ergosterol index was estimated as an indicator of membrane composition and conidial physiological status. Conidial suspensions were prepared as described above for melanin extraction and subsequently dried at 60 °C. Ergosterol extraction was performed by adding 1.5 mL of 25% (*w*/*v*) alcoholic KOH solution, followed by incubation at 85 °C for 1 h. After digestion, 1 mL of distilled water and 1.5 mL of n-heptane were added, and the mixture was vortexed for 5 min and incubated at −20 °C for 18 h. The organic phase was then recovered and mixed with four volumes of absolute ethanol. After homogenization, absorbance was measured at 230 and 281.5 nm. A relative ergosterol index was calculated according to the method described by Breivik and Owades (1957) [28], using the absorbance values at both wavelengths and normalizing the result by the log_10_ of the conidial count. This index was used exclusively for comparative analysis among treatments and does not represent an analytically validated absolute ergosterol concentration.

#### 2.2.3. Functional Characterization: Tolerance to Thermal Stress

Thermotolerance was evaluated as a functional indicator of conidial quality based on germination after heat exposure. Aliquots containing 1 mL of conidial suspension at a concentration of 1 × 10^7^ conidia mL^−1^ were exposed to 50 °C for 1 h, whereas non-heat-treated aliquots maintained at room temperature (RT) were used as controls (modified from [29]). Following heat treatment, conidial germination was assessed on water agar. Five 5 µL aliquots of each conidial suspension were inoculated onto water agar plates as technical replicates and incubated at 28 °C for 18–20 h. After incubation, agar fragments containing the inoculated conidia were examined microscopically, and a minimum of 100 conidia (germinated and non-germinated) were counted per aliquot. Conidia were considered germinated when the germ tube length was equal to or greater than the conidial diameter. Germination percentage was calculated as the proportion of germinated conidia relative to the total number of conidia counted. The assay was performed using five independent biological replicates per treatment.

### 2.3. Conidiation-Related Gene Expression

To complement the characterization of conidia phenotypic traits, the expression of selected genes involved in conidiation (*brlA*, *abaA*, *fluG* and *rodA*) was evaluated. Putative orthologs of these genes were identified in *Talaromyces sayulitensis* HC1 by PCR amplification using primers designed from homologous sequences of *Talaromyces marneffei* ATCC 18224 and *Talaromyces stipitatus* ATCC 10500 (Table 2). Amplified fragments were sequenced, edited using CLC Main Workbench 7 and compared against public databases using BLAST [30].

For gene expression analysis, samples were collected after 2, 4, 6 and 8 days of culture. Total RNA was extracted using TRIzol reagent (Invitrogen, Carlsbad, CA, USA) following cell lysis, and RNA integrity was verified by agarose gel electrophoresis. First-strand cDNA synthesis was performed using the Transcriptor First Strand cDNA Synthesis Kit (Roche Diagnostics, Mannheim, Germany) according to the manufacturer’s instructions.

Quantitative real-time PCR (RT-qPCR) was performed using a LightCycler^®^ 480 System (Roche Diagnostics Ltd., Rotkreuz, Switzerland) and SYBR Green chemistry. Reaction mixtures (10 μL) contained 5 μL of LightCycler^®^ 480 SYBR Green I Master Mix, 1 μL of each primer (2.5 μM), 2 μL of cDNA (5 ng μL^−1^) and nuclease-free water. Amplification was carried out with an initial denaturation at 95 °C for 5 min, followed by 45 cycles of 95 °C for 10 s, 56 °C for 15 s and 72 °C for 15 s. Melting curve analysis was performed from 65 °C to 97 °C to verify amplification specificity. Samples from each biological replicate were analyzed in technical triplicate, and no-reverse-transcriptase controls were included to exclude genomic DNA contamination. Relative gene expression was calculated according to the Pfaffl method [31] using the Relative Expression Software Tool (REST) [32], with *act1* and *tub1* as reference genes. For data visualization, normalized relative quantity (NRQ) values were transformed to log10 scale prior to graphical representation. Expression levels were compared between culture systems and culture medium compositions.

### 2.4. Statistical Analysis

Data were analyzed after verification of normality and homogeneity of variance assumptions. Differences among treatments were evaluated by one-way analysis of variance (ANOVA), followed by Tukey’s multiple comparison test when significant differences were detected. In addition, the effects of culture system (solid-state versus submerged fermentation) and culture medium composition (two culture media with different compositions), as well as their interaction, were evaluated using two-way ANOVA. Pearson correlation analysis was performed to evaluate associations among morphological, physicochemical, physiological and molecular variables. Correlation patterns were visualized by hierarchical clustering using a Pearson correlation heat map generated with MetaboAnalyst 6.0. Statistical significance was established at *p* < 0.05.

### 2.5. Artificial Intelligence Statement

Generative artificial intelligence (ChatGPT-5.5, OpenAI, San Francisco, CA, USA) was used to assist in drafting, editing, language refinement, organization, and visualization of portions of the manuscript. The tool also assisted in improving clarity and readability and in supporting the presentation and discussion of scientific results. Artificial intelligence was not used to generate experimental data, perform statistical analyses, or independently interpret scientific findings. All statistical analyses were conducted using the software described in the Section 2, and all scientific interpretations, conclusions, and final decisions regarding the manuscript were made, reviewed, and approved by the authors, who take full responsibility for the content of the article.

## 3. Results

The effects of culture system (solid-state or submerged fermentation) and culture medium composition on the quality of *Talaromyces sayulitensis* HC1 conidia were evaluated through the characterization of morphological, physicochemical and functional attributes. In addition, the expression of selected conidiation-related genes was analyzed to assess molecular responses to different culture conditions.

### 3.1. Effects of Culture Conditions on Conidial Morphology

**Figure 1:**
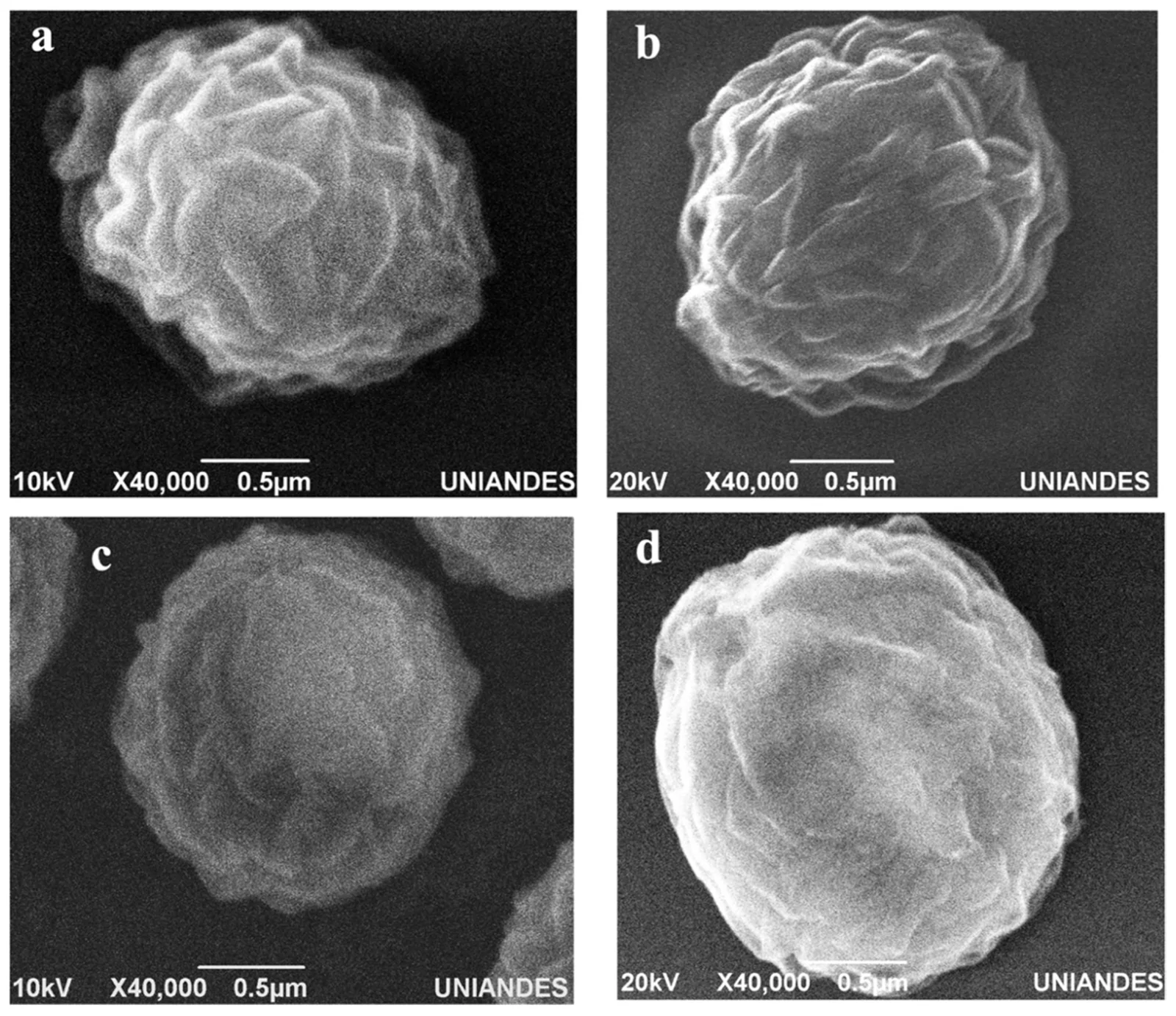
Scanning electron micrographs showing the surface morphology of *Talaromyces sayulitensis* HC1 conidia produced under different culture conditions. (**a**) S10-SSF, (**b**) S10-SmF, (**c**) S5-SSF and (**d**) S5-SmF. All treatments exhibited a striated surface ornamentation characterized by irregularly arranged ridges. Scale bars = 0.5 μm.

**Figure 2:**
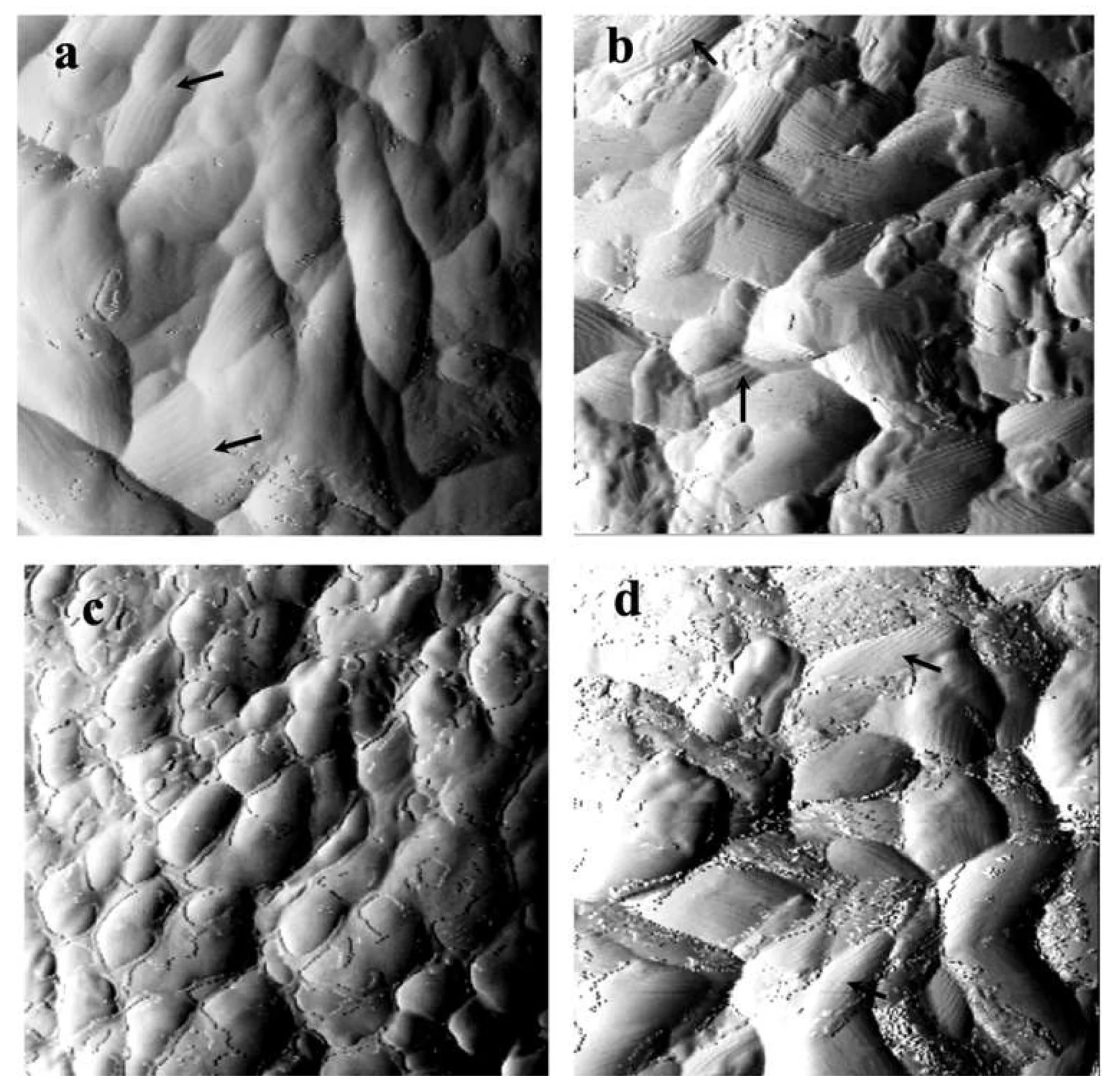
Atomic force microscopy images showing the surface topography of *Talaromyces sayulitensis* HC1 conidia produced under different culture conditions. (**a**) S10-SSF, (**b**) S10-SmF, (**c**) S5-SSF and (**d**) S5-SmF. Arrows indicate surface ridges exhibiting a rodlet-like organization, which was observed under all culture conditions. Scan area: 1 × 1 μm.

Conidial morphology differed according to the culture system but not according to the culture medium composition (Figure 1, Table 3). Conidia produced under solid-state fermentation (SSF) were predominantly cylindrical, with circularity index values of 0.927 ± 0.108 and 0.901 ± 0.092 for S10-SSF and S5-SSF, respectively. In contrast, conidia produced under submerged fermentation (SmF) displayed a more ellipsoidal morphology, with lower circularity values (0.860 ± 0.102 and 0.868 ± 0.101 for S10-SmF and S5-SmF, respectively). Conidia obtained under SmF were also larger than those produced under SSF, exhibiting average projected areas of 3.609 ± 0.727 and 3.754 ± 0.503 μm^2^, compared with 2.911 ± 0.367 and 2.877 ± 0.233 μm^2^ for S10-SSF and S5-SSF, respectively (Table 3). In contrast, culture medium composition did not significantly affect conidial size or circularity in either culture system. Despite these differences in size and shape, all conidia exhibited a striate surface ornamentation characterized by low, irregularly arranged ridges (Figure 1). AFM analysis further revealed the presence of rodlet-like structures on the conidial surface under all culture conditions (Figure 2).

**Figure 3:**
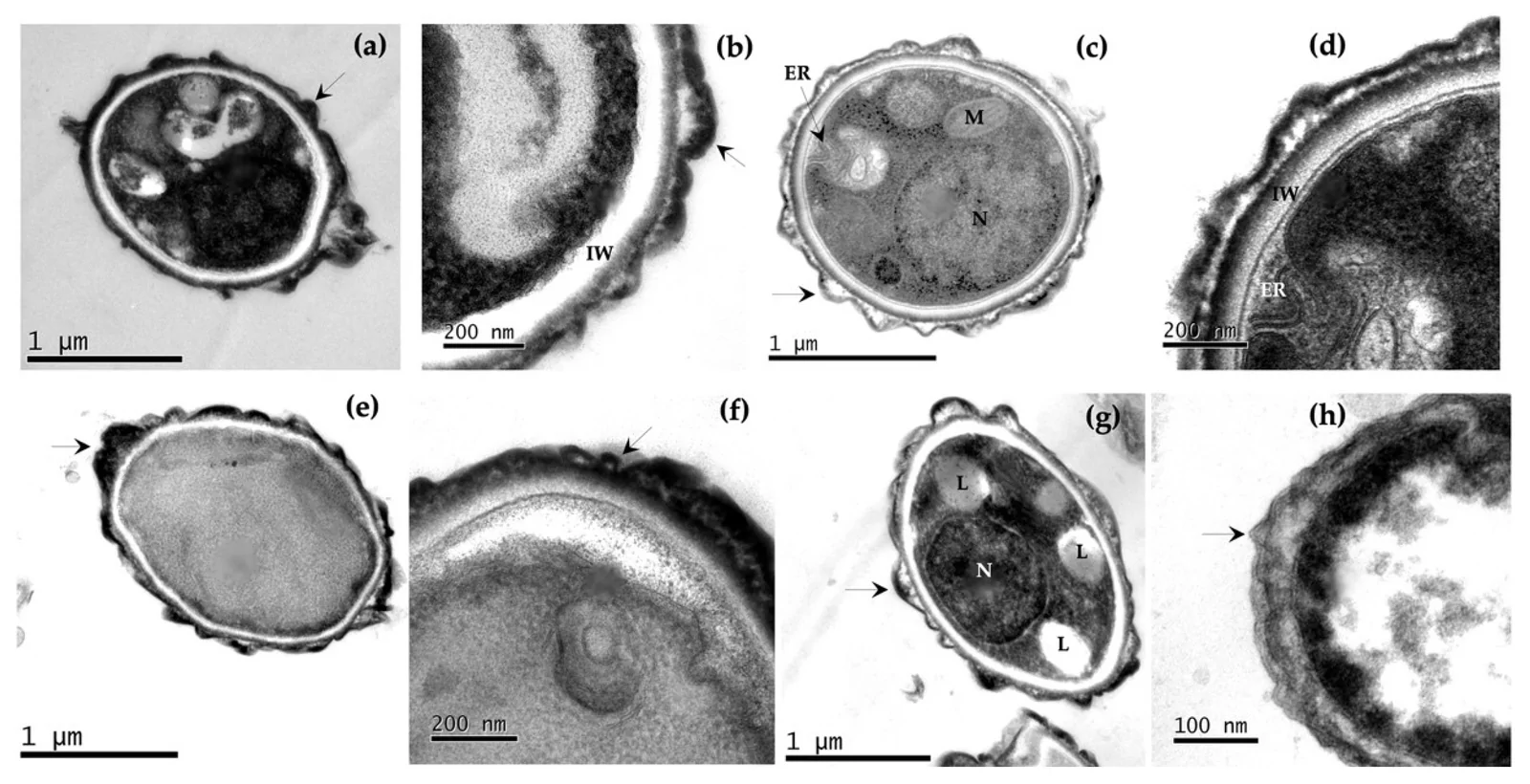
Transmission electron micrographs showing the ultrastructure and cell wall architecture of *Talaromyces sayulitensis* HC1 conidia produced under different culture conditions. (**a**,**b**) S10-SSF, (**c**,**d**) S10-SmF, (**e**,**f**) S5-SSF and (**g**,**h**) S5-SmF. M: mitochondrion; N: nucleus; L: lipid body; ER: membrane profiles consistent with endoplasmic reticulum. Arrows indicate the electron-dense outer cell wall layer, whereas IW denotes the inner wall layer.

Transmission electron microscopy revealed that conidia produced under all culture conditions exhibited a typical eukaryotic cellular organization, including cytoplasmic organelles enclosed by a cell wall composed of two layers with distinct electron densities (Figure 3). The outer cell wall layer was highly electron-dense and exhibited considerable variation in thickness among culture conditions. Conidia produced in S10-SSF, S10-SmF and S5-SSF displayed similar outer wall thickness values (84.3 ± 36.3, 68.2 ± 30.9 and 75.7 ± 31.9 nm, respectively), whereas conidia produced in S5-SmF exhibited a significantly thinner outer wall layer (20.9 ± 6.8 nm; *p* < 0.05) (Table 3). In contrast, the inner cell wall layer exhibited a relatively uniform thickness and consistently lower electron density across all treatments.

To determine whether the observed morphological and ultrastructural differences affected the mechanical properties of the conidial wall, Young’s modulus (E) was measured by AFM. Values ranged from 222.802 ± 234.692 MPa for S5-SSF conidia to 439.976 ± 211.824 MPa for S5-SmF conidia. However, no statistically significant differences were detected in surface nanomechanical stiffness among culture conditions (Table 3), despite the observed differences in conidial morphology and outer cell wall thickness.

### 3.2. Effects of Culture Conditions on Conidial Physicochemical Properties

**Figure 4:**
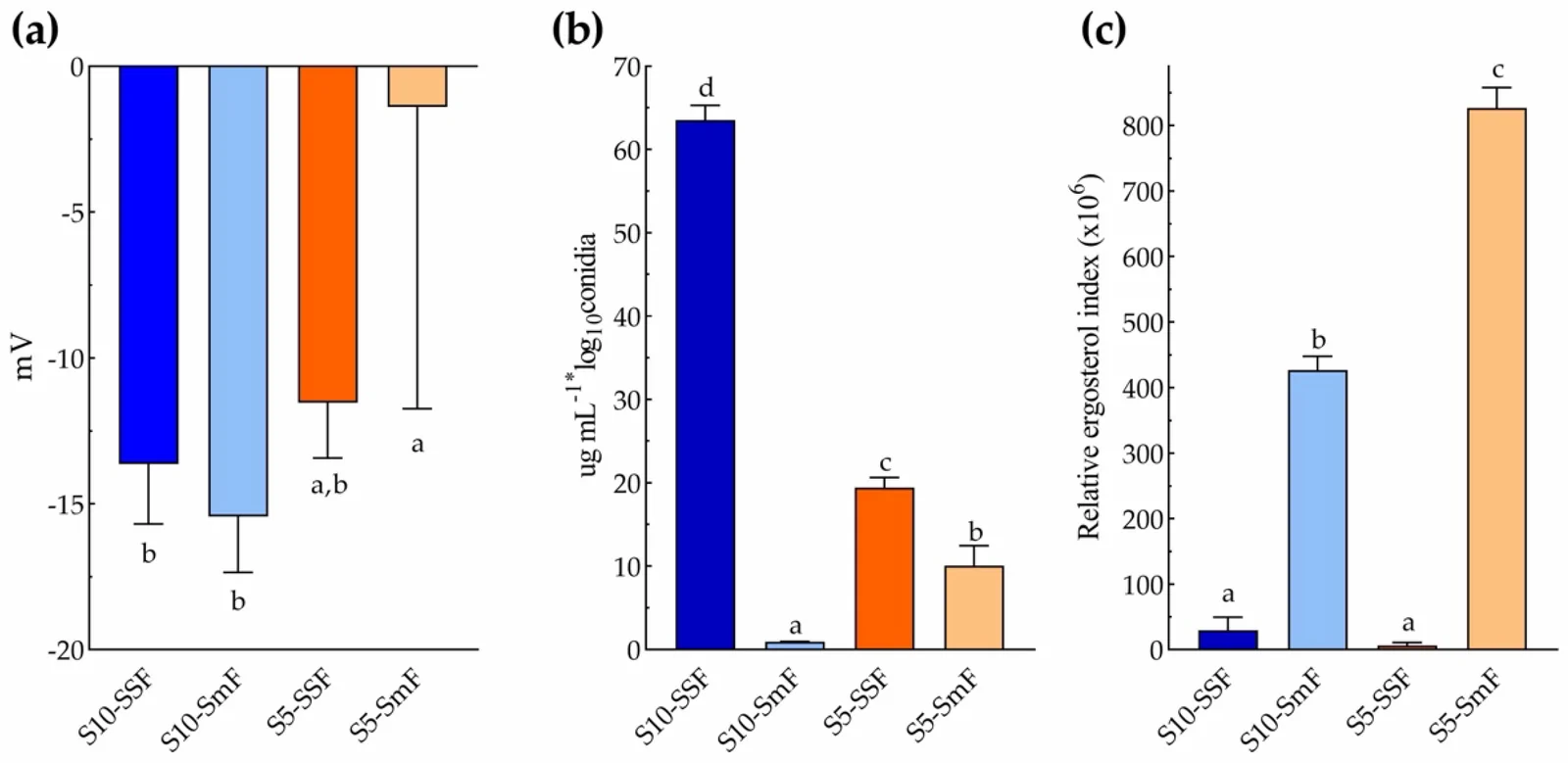
Effect of culture conditions on physicochemical properties of *Talaromyces sayulitensis* HC1 conidia. (**a**) Zeta potential. (**b**) Melanin content. (**c**) Relative ergosterol index (×10^6^). Data are presented as mean ± standard deviation. Different letters indicate statistically significant differences among treatments (*p* < 0.05).

Conidia produced under all culture conditions exhibited a negative zeta potential, indicating a predominantly electronegative surface (Figure 4a). However, surface electrostatic properties were significantly influenced by culture medium composition. The most negative zeta potential values were observed in S10-derived conidia (S10-SSF, −13.63 ± 2.05 mV; S10-SmF, −15.43 ± 1.91 mV), whereas S5-derived conidia exhibited significantly less negative zeta potential values (*p* < 0.05). The least negative values were observed in S5-SmF conidia (−1.39 ± 10.35 mV), although considerable variability was detected in this treatment.

The physicochemical composition of conidia was also affected by culture conditions. Both culture system (F = 2851.94, *p* < 0.001) and culture medium composition (F = 673.23, *p* < 0.001) significantly influenced melanin accumulation, with culture system exerting the strongest effect. Conidia produced under SSF accumulated substantially higher melanin levels (S10-SSF, 63.53 ± 1.78 μg mL^−1^·log_10_ conidia^−1^; S5-SSF, 19.43 ± 1.22 μg mL^−1^·log_10_ conidia^−1^) than those produced under SmF (S10-SmF, 0.92 ± 0.05 μg mL^−1^·log_10_ conidia^−1^; S5-SmF, 10.06 ± 2.42 μg mL^−1^·log_10_ conidia^−1^) (Figure 4b).

The relative ergosterol index (×10^6^) showed a contrasting pattern. Both culture system and culture medium composition significantly affected relative ergosterol accumulation (*p* < 0.001), with the highest values observed in conidia produced under submerged fermentation, particularly in S5-SmF (826.4 ± 31.0), followed by S10-SmF (426.4 ± 21.3). In contrast, substantially lower values were detected in SSF-derived conidia (S10-SSF, 29.5 ± 20.0; S5-SSF, 6.4 ± 4.5) (Figure 4c).

Overall, distinct physicochemical profiles were observed among culture conditions, with higher melanin accumulation in SSF-derived conidia and higher relative ergosterol levels in SmF-derived conidia.

### 3.3. Effects of Culture Conditions on Conidial Germination and Thermotolerance

**Figure 5:**
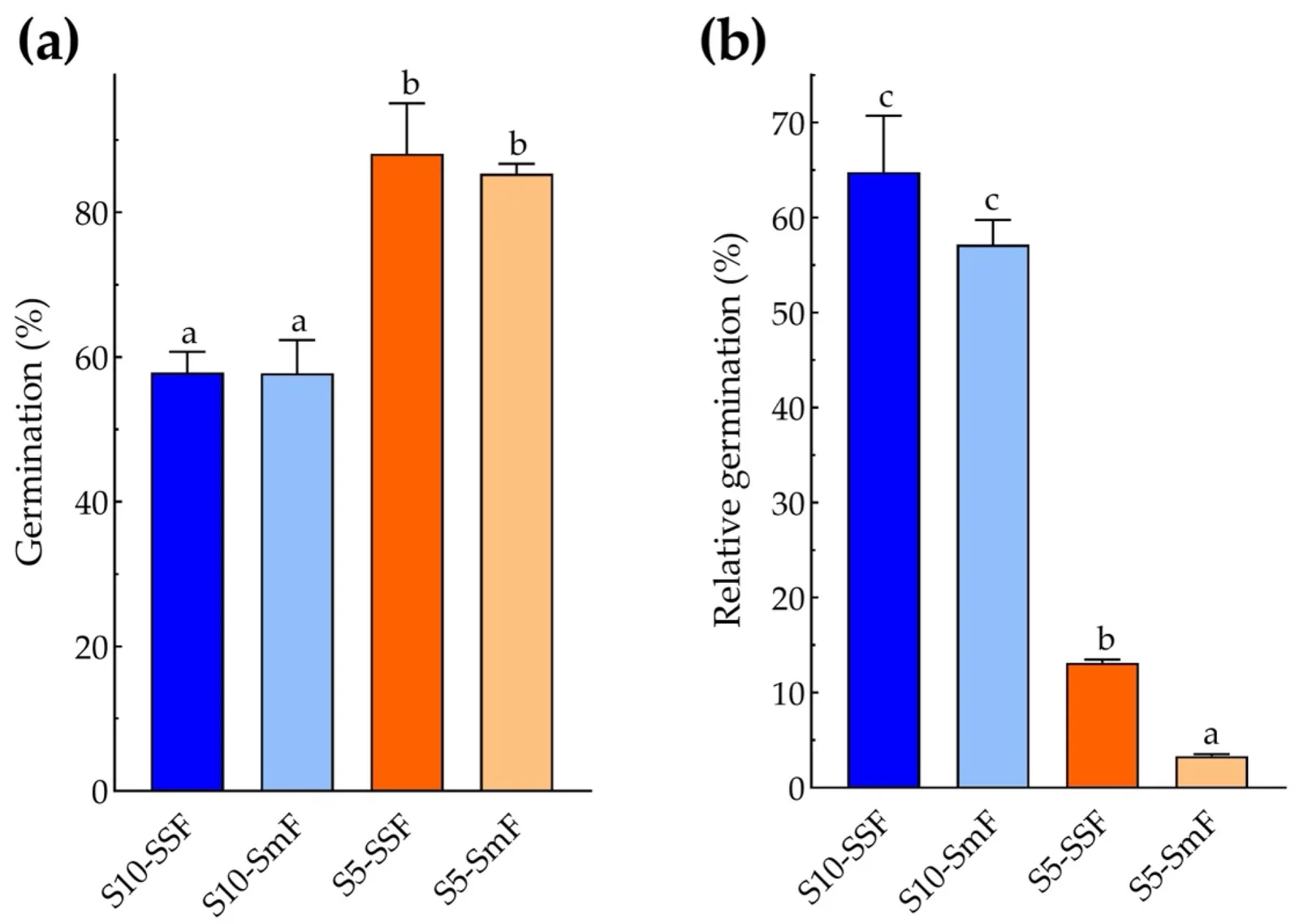
Effect of culture conditions on conidial germination and thermotolerance *of Talaromyces sayulitensis* HC1. (**a**) Germination percentage of conidia produced under solid-state fermentation (SSF) and submerged fermentation (SmF) using the two media evaluated (S10 and S5). (**b**) Relative germination after thermal stress, used as an indicator of conidial thermotolerance. Data are presented as mean ± standard deviation. Different letters indicate statistically significant differences among treatments (*p* < 0.05).

Culture conditions significantly affected both conidial germination and thermotolerance (Figure 5). For germination, culture medium composition significantly affected germination capacity (F = 19.56, *p* < 0.0001), whereas the culture system (SSF versus SmF) did not have a significant effect. Conidia produced using the S5 culture medium composition exhibited significantly higher germination percentages than those produced using the S10 culture medium composition, irrespective of the culture system. Germination values reached 85.37 ± 0.79% and 83.66 ± 2.85% for S5-SSF and S5-SmF, respectively, compared with 57.86 ± 1.67% and 56.47 ± 4.03% for S10-SSF and S10-SmF (Figure 5a).

In contrast, thermotolerance showed the opposite trend (Figure 5b). Both culture medium composition (F = 799.8, *p* < 0.001) and culture system (F = 21.99, *p* < 0.001) significantly affected thermotolerance. The highest relative germination values after thermal stress were observed in S10-derived conidia, reaching 64.77 ± 5.91% and 57.15 ± 2.57% for S10-SSF and S10-SmF, respectively. In contrast, S5-derived conidia exhibited markedly lower thermotolerance, yielding relative germination values of 13.17 ± 0.34% for S5-SSF and 3.30 ± 0.21% for S5-SmF.

These results highlight contrasting effects of culture medium composition on conidial performance: S5-derived conidia exhibited higher germination percentages, whereas S10-derived conidia exhibited greater thermotolerance.

### 3.4. Identification of the brlA, abaA, fluG, and rodA Orthologs in T. sayulitensis

BLAST analysis of the consensus sequences obtained for *brlA*, *abaA*, *fluG*, and *rodA* was performed using the Basic Local Alignment Search Tool (BLAST) available through the National Center for Biotechnology Information (NCBI). Sequence identities ranging from 67 to 91% with homologous genes previously reported in other filamentous fungi. For *brlA*, the highest sequence identities were observed with sequences from *Talaromyces marneffei* (90–91%) and *Talaromyces stipitatus* (88%). For *abaA*, the highest similarities were 87% and 82% with the same species. *fluG* exhibited identities ranging from 72 to 89%, whereas *rodA* showed identities between 80 and 91% with homologous sequences from *Talaromyces* and *Penicillium* species. These sequence similarities are consistent with the identification of the amplified fragments as putative orthologs of the target conidiation-related genes in *T. sayulitensis* HC1.

Based on the identified sequences, primers were designed (Table 2) to assess the differential expression of *brlA*, *abaA*, *fluG*, and *rodA* under different culture conditions. Primer specificity was verified by conventional PCR, and amplification efficiencies were calculated for RT-qPCR analysis.

### 3.5. Relative Expression of brlA, abaA, fluG, and rodA Under Different Culture Systems and Culture Medium Compositions

**Figure 6:**
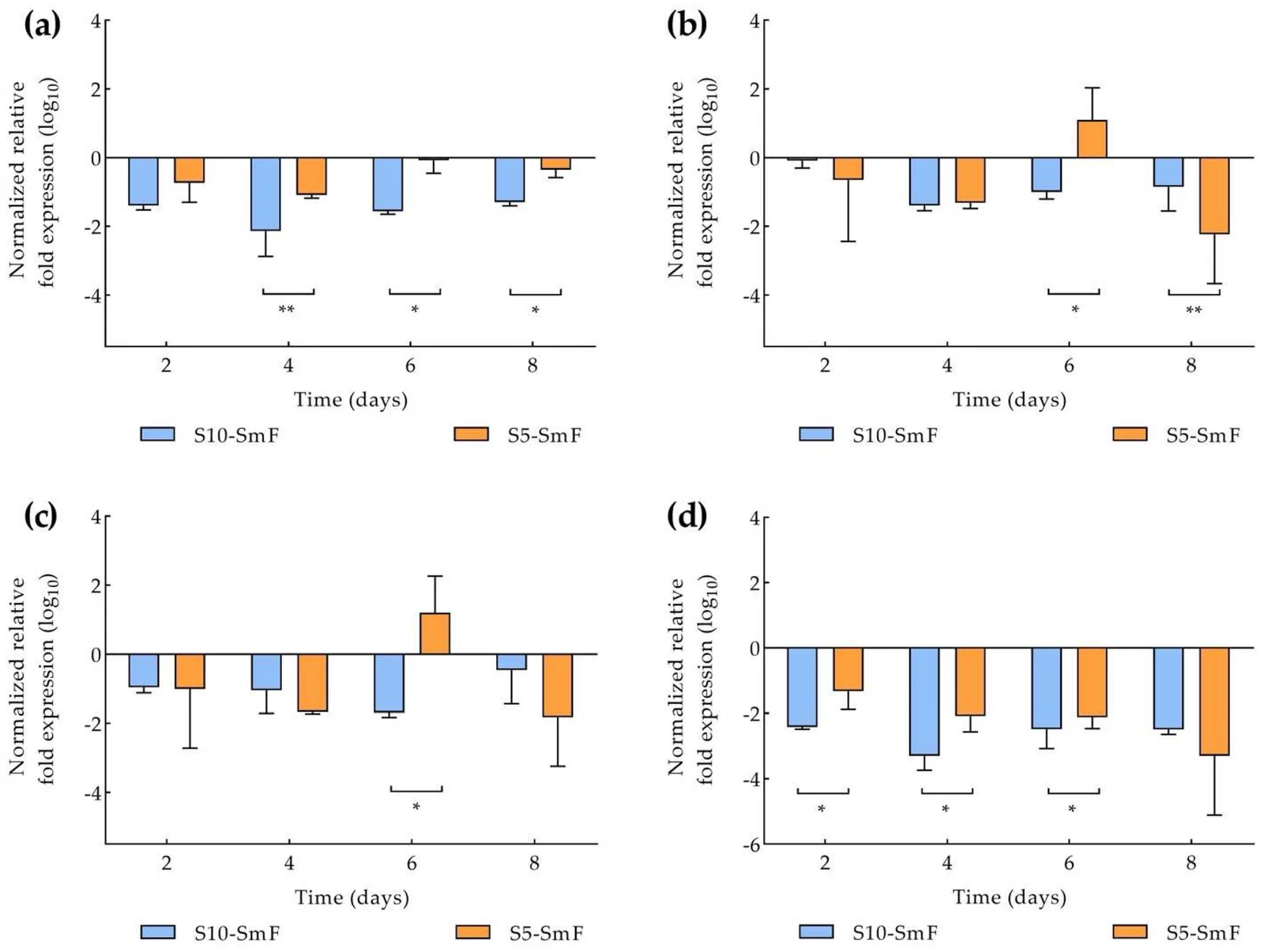
Relative expression of conidiation-related genes in cultures grown under submerged fermentation (SmF) relative to the corresponding solid-state fermentation (SSF) cultures for each of the two culture medium compositions evaluated. (**a**) *brlA*, (**b**) *abaA*, (**c**) *fluG* and (**d**) *rodA*. Expression levels were normalized using act1 and tub1 as reference genes and calculated according to the Pfaffl method. Values are expressed as log_10_-transformed normalized relative quantity (NRQ). Asterisks indicate statistically significant differences between SmF and the corresponding SSF cultures at each sampling time (* *p* < 0.05; ** *p* < 0.01).

To assess how culture system and culture medium composition affect the expression of genes involved in conidiation, the expression of *brlA*, *abaA*, *fluG*, and *rodA* was analyzed at 2, 4, 6, and 8 days of culture (Section 2.3). Comparisons between culture systems are presented in Figure 6, whereas the effect of culture medium composition is shown in Supplementary Figure S3.

Overall, *brlA*, *abaA*, *fluG*, and *rodA* exhibited lower expression levels in submerged cultures than in the corresponding solid-state cultures at most sampling times. Two exceptions were observed for *abaA* and *fluG* on day 6, when expression levels in submerged cultures grown using the S5 culture medium composition approached or exceeded those observed under the corresponding solid-state conditions.

Differences associated with culture medium composition were also observed. In general, expression levels remained higher in submerged cultures grown using the S5 culture medium composition than in the corresponding S10 cultures, although the magnitude and timing of this response varied among genes. For the *brlA* gene, this difference was significant on days 4 (*p* < 0.01), 6 (*p* < 0.05), and 8 (*p* < 0.05) (Figure 6a). For *abaA*, significant differences were observed on days 6 (*p* < 0.05) and 8 (*p* < 0.01) (Figure 6b). For *fluG*, this difference was significant only on day 6 (*p* < 0.05) (Figure 6c). Lastly, for *rodA*, significant differences were detected on days 2, 4, and 6 (*p* < 0.05) (Figure 6d).

### 3.6. Integrated Correlation Analysis of Variables Associated with Conidial Quality

**Figure 7:**
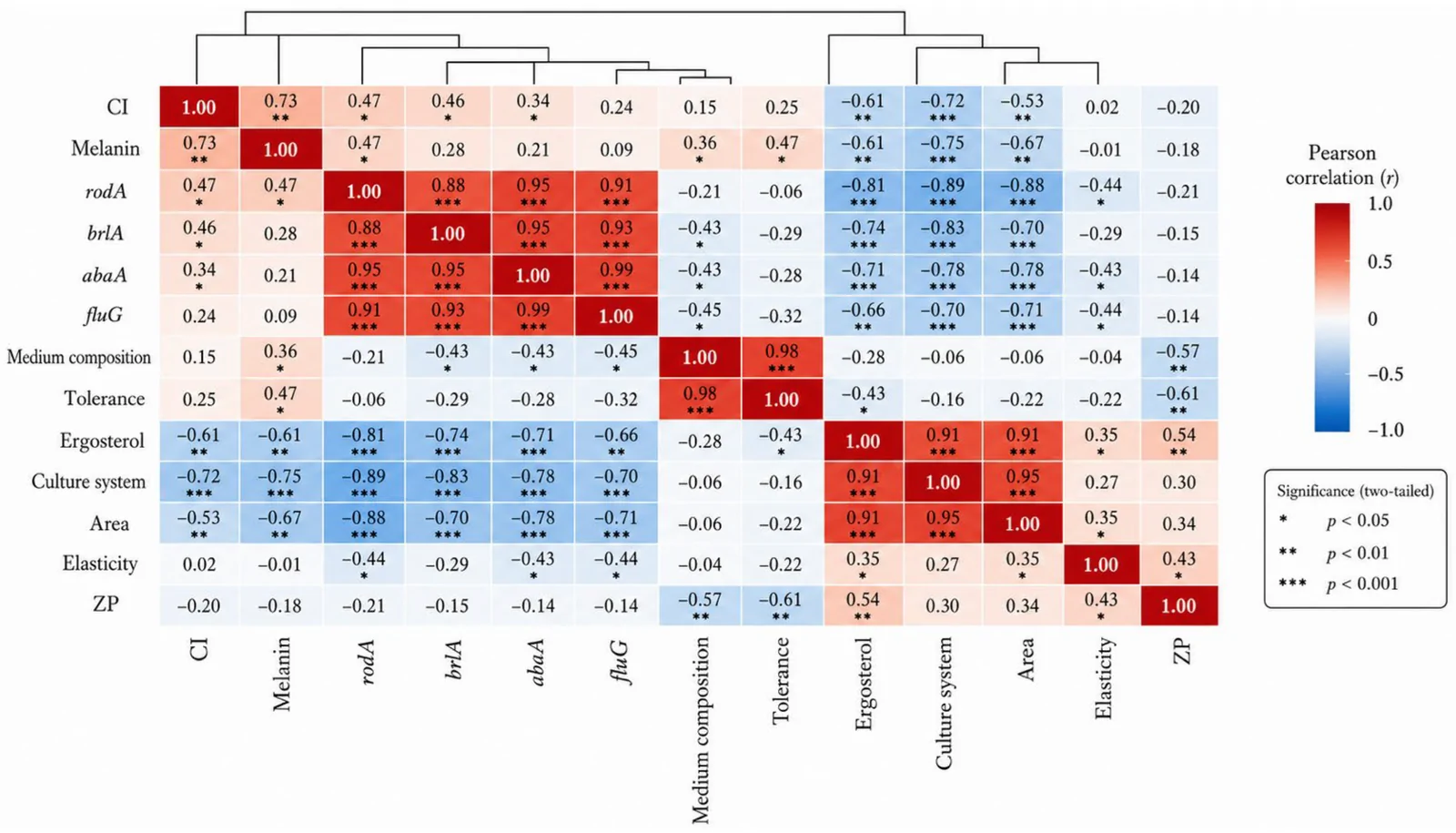
Pearson correlation heat map showing the relationships among morphological, physicochemical, and physiological traits of *Talaromyces sayulitensis* HC1 conidia and the expression of selected genes involved in conidiogenesis. Variables were hierarchically clustered according to their Pearson correlation profiles. Positive correlations are shown in red and negative correlations in blue, with color intensity proportional to the Pearson correlation coefficient (r). Culture system indicates the fermentation system under which the conidia were produced (solid-state fermentation, SSF, or submerged fermentation, SmF). Culture medium composition indicates the culture medium used during conidial production (S5 or S10). CI, circularity index; *brlA*, *abaA*, *fluG*, and *rodA*, genes involved in conidiogenesis; ZP, zeta potential.

To explore the relationships among the variables evaluated in this study, Pearson correlation analysis followed by hierarchical clustering was performed using morphological, physicochemical, and physiological conidial traits together with the expression of selected conidiation-related genes and culture-related variables (Figure 7). Hierarchical clustering identified four major groups of associated variables. The conidiation-related genes (*rodA*, *brlA*, *abaA*, and *fluG*) clustered together and showed predominantly positive correlations with melanin content and the conidial circularity index (CI). In contrast, the culture system, conidial area, and ergosterol content formed a second cluster that was negatively correlated with the conidiation-related genes. A third cluster grouped culture medium composition with thermotolerance, whereas elasticity and zeta potential (ZP) exhibited weaker associations with the remaining variables. Overall, the evaluated variables exhibited distinct patterns of positive and negative correlations across the different culture conditions.

## 4. Discussion

### 4.1. Culture System Shapes the Conidial Phenotype

Culture system had a marked effect on the phenotype of *Talaromyces sayulitensis* HC1 conidia. Conidia produced under SSF were smaller, more cylindrical and accumulated substantially higher levels of melanin than those produced under SmF, whereas conidia produced under SmF exhibited higher relative ergosterol content. In contrast, the thickness of the outer cell wall was influenced by both the culture system and culture medium composition, indicating that this trait cannot be attributed exclusively to the culture system. Together, these differences demonstrate that both culture system and culture medium composition contribute to shaping the conidial phenotype, although their effects vary among individual traits.

Several studies have proposed that SSF and related solid-culture systems provide environmental conditions that more closely resemble the natural habitat of many filamentous fungi than SmF. Because the evolutionary diversification of many fungal lineages has occurred in association with moist solid substrates and air-exposed surfaces, submerged culture may represent a less ecologically representative environment that can modify fungal physiology and development [5,8]. In SSF or solid-medium culture, fungi are exposed to solid–air interfaces, gradients of water activity, nutrient availability and oxygen concentration, which are absent or less pronounced in liquid cultures. These observations support the concept of a distinct “solid-medium physiology”, in which fungi exhibit physiological and developmental responses that differ substantially from those observed under submerged culture conditions [5,33].

The morphological, ultrastructural and physicochemical differences observed in the present study are consistent with this concept. Conidia produced under SSF displayed greater melanin accumulation than those obtained under SmF, whereas conidia produced under SmF contained higher relative amounts of ergosterol. In contrast, the thickness of the outer cell wall varied according to both the culture system and the culture medium composition evaluated. Melanin, ergosterol and cell wall architecture represent different structural components of the conidial phenotype and are associated with distinct biological functions. Whereas melanin contributes primarily to environmental protection, ergosterol is an essential membrane sterol involved in membrane organization and fluidity, and cell wall remodeling is a characteristic feature of conidial development. For example, studies in the filamentous fungi *Pseudallescheria boydii*, *Beauveria bassiana* and *Metarhizium robertsii* have shown that conidial development is accompanied by progressive changes in cell wall architecture, pigmentation, surface properties and stress tolerance [34,35,36]. Therefore, the phenotypic differences observed between SSF and SmF suggest that the culture system modulates multiple structural components of the conidium rather than a single characteristic. This interpretation is further supported by the correlation analysis, which grouped the expression of the selected conidiation-related genes together with melanin content and conidial circularity. Although this association does not imply a causal relationship, it indicates that the expression of these developmental genes was associated with conidial traits that developed under the same culture conditions.

The AFM and SEM analyses further support this interpretation. Rodlet-like surface structures were observed on conidia produced under all culture conditions, which is consistent with, but does not confirm, the presence of hydrophobin-rich surface layers. Hydrophobins are major components of the rodlet layer and contribute to conidial surface hydrophobicity, adhesion and other biological functions associated with fungal development and environmental interactions [14]. In addition, the abundance of hydrophobin-related surface proteins has been proposed as a useful trait for selecting fungal propagules for biocontrol formulation development [14]. Although rodlet-like structures were observed under all culture conditions, their abundance and protein composition were not quantified; therefore, possible differences in the rodlet layer among culture conditions could not be determined. Nevertheless, the increased melanization and development of thicker outer wall layers observed under specific culture conditions support the existence of distinct surface phenotypes.

The differences observed between SSF and SmF may also be related to broader physiological changes induced by growth under solid-state or solid-medium conditions. Comparative studies have shown that fungi cultivated under SSF can exhibit greater secretome complexity, altered protein secretion patterns and reduced catabolite repression relative to SmF cultures [37]. Likewise, in *Neurospora sitophila*, growth under SSF was associated with greater biomass production and increased secretion of proteins related to mycelial growth and conidiation, whereas SmF cultures were enriched in proteins associated with glycolytic metabolism and stress tolerance [38]. Consistent with these observations, combined transcriptomic and metabolomic analyses in *Penicillium expansum* revealed culture-system-dependent profiles of secondary metabolism and differential expression of ROS and defense related genes under SSF and SmF [39]. Together, these observations indicate that solid-state or solid-medium culture conditions can induce physiological and developmental responses distinct from those observed under submerged conditions.

The generally higher expression of *brlA*, *abaA*, *fluG* and *rodA* observed under SSF provides molecular evidence that the culture system influenced selected genes associated with conidiation and conidial development. Previous studies in filamentous fungi have shown that *brlA, abaA* and *fluG* participate in regulatory networks controlling conidiation and conidial differentiation [20,40]. In addition, studies in *Penicillium camemberti* demonstrated that *rodA* expression is associated with conidiation under solid culture conditions and with the formation of hydrophobic surface structures [41]. A subsequent study in the same species found no significant differences in the temporal expression patterns of *brlA* and *wetA* among the evaluated solid and liquid culture conditions, whereas *rodA* expression was lower or delayed in liquid culture; this pattern coincided with lower hydrophobicity and reduced survival during storage of liquid-culture-derived conidia [42]. These findings indicate that individual components of the conidiation regulatory network may respond differently to the culture system. However, because the present molecular analysis was limited to four selected genes, our results should be interpreted as complementary evidence rather than as a comprehensive characterization of the regulatory response to SSF and SmF.

Overall, the results demonstrate that the culture system markedly influenced the conidial phenotype by modifying multiple morphological, ultrastructural and physicochemical attributes, as well as the expression of selected conidiation-related genes. The integrated correlation analysis further supported this interpretation by revealing associations among the evaluated morphological, physicochemical and molecular variables under the different culture conditions. Nevertheless, as discussed below, the functional consequences of these phenotypic differences were strongly influenced by the culture medium composition evaluated in this study.

### 4.2. Culture Medium Composition Differentially Affects Conidial Germination and Thermotolerance

The results obtained in the present study indicate that culture medium composition during conidiation strongly influenced the functional attributes of *Talaromyces sayulitensis* HC1 conidia. In this work, the evaluated media differed in sucrose and ammonium phosphate concentrations and, consequently, in their C:N ratios, making it impossible to distinguish the individual contribution of these variables. Therefore, throughout this discussion, “culture medium composition” refers to the combined effect of the changes in sucrose and ammonium phosphate concentrations and the resulting change in C:N ratio. Conidia produced across both culture systems (SSF and SmF) using the medium composition containing a lower sucrose concentration and a higher ammonium phosphate concentration (C:N ratio 10:1) showed increased germination capacity but reduced thermotolerance compared with those produced using the medium composition containing a higher sucrose concentration and a lower ammonium phosphate concentration (C:N ratio 50:1) (Figure 5). These findings indicate that different components of conidial quality respond differently to the culture medium composition used during conidiation, emphasizing that improvements in one functional attribute do not necessarily translate into improvements in others.

The effects of culture medium composition on fungal propagules have been widely documented, influencing not only the quantity of propagules produced but also their physiological characteristics and biological performance. Depending on the species and experimental conditions, these responses may be associated with the source or concentration of carbon, the source or concentration of nitrogen, the resulting C:N ratio, or their combined effects. However, the direction and magnitude of these effects are often species-dependent, reflecting differences in metabolic regulation and developmental strategies among filamentous fungi [43,44,45]. In submerged cultures of *Penicillium camemberti*, conidiation depended on the nitrogen source, occurring in a synthetic medium containing KNO_3_ but being inhibited by the complex nitrogen sources evaluated [42]. More recently, transcriptomic analysis in the same species showed that conidial production also depended on carbon source and concentration: conidiation occurred with 10 g/L glucose but not with 20 g/L glucose or with sucrose under the evaluated conditions [46]. Consistent with this concept, the culture medium compositions evaluated in the present study had contrasting effects on conidial production depending on the culture system. Under SmF conditions, the medium composition containing a lower sucrose concentration and a higher ammonium phosphate concentration (C:N ratio 10:1) increased conidial production, whereas under SSF the same culture medium composition caused a slight but significant reduction in conidial yield (Supplementary Figure S2). These observations suggest that the effect of culture medium composition on conidiation depends on the physical culture environment and may influence the balance between vegetative growth and conidiation.

Despite these differences in conidial production, culture medium composition had a remarkably consistent effect on germination. Conidia produced across both culture systems (SSF and SmF) using the medium composition containing a lower sucrose concentration and a higher ammonium phosphate concentration (C:N ratio 10:1) exhibited significantly higher germination percentages than those produced using the medium composition containing a higher sucrose concentration and a lower ammonium phosphate concentration (C:N ratio 50:1). Similar observations have been reported in other filamentous fungi. In *Colletotrichum truncatum*, conidia produced at lower C:N ratios germinated more rapidly and displayed greater biological efficacy than conidia produced at higher C:N ratios [43]. Likewise, conidia of *Colletotrichum coccodes* produced under relatively low C:N ratios exhibited high germination rates and greater biological efficacy, whereas increasing the C:N ratio reduced germination, conidiation and overall conidial quality [44]. Comparable results were obtained in *Trichoderma atroviride*, where nutritional amendments and relatively low C:N ratios enhanced conidial germination and bioactivity [47]. Together, these studies support the idea that culture medium composition during conidiation strongly influences the physiological state of fungal propagules and their capacity to resume growth. The integrated correlation analysis further supports this interpretation by identifying a close association between culture medium composition and thermotolerance, reinforcing the concept that different functional attributes of conidia may respond independently to the culture medium composition used during conidiation.

Several mechanisms have been proposed to explain these responses. Culture medium composition during conidiation can influence reserve accumulation and the physiological state of fungal propagules. In *Colletotrichum truncatum*, conidia produced at low C:N ratios accumulated higher protein contents and lower lipid contents than conidia produced at higher C:N ratios [43]. These conidia also germinated more rapidly and exhibited greater biological efficacy, suggesting that reserve composition may influence the physiological readiness of propagules to resume growth. Therefore, the increased germination observed in the present study in conidia produced using the medium composition containing a lower sucrose concentration and a higher ammonium phosphate concentration (C:N ratio 10:1) is consistent with previous evidence indicating that culture medium composition during conidiation affects the physiological state of conidia and their capacity to initiate growth. However, because conidial reserves were not quantified in the present study, their contribution to the observed differences in germination remains to be determined.

In contrast to germination, thermotolerance showed the opposite response. Conidia produced using the medium composition containing a higher sucrose concentration and a lower ammonium phosphate concentration (C:N ratio 50:1) exhibited substantially greater relative germination after thermal stress than conidia produced using the medium composition containing a lower sucrose concentration and a higher ammonium phosphate concentration (C:N ratio 10:1). This pattern was observed in both culture systems and suggests that the mechanisms promoting rapid germination are not necessarily the same as those conferring resistance to environmental stress. The contrasting responses of these two traits suggest that different components of conidial quality are not necessarily coupled and may respond differently to culture medium composition during conidiation.

The phenotypic differences identified in this study provide a possible explanation for this contrasting behavior. As discussed in Section 4.1, conidia produced using the culture medium composition containing a higher sucrose concentration and a lower ammonium phosphate concentration (C:N ratio 50:1) exhibited greater melanization and, in some comparisons, developed thicker outer cell wall layers, traits commonly associated with enhanced protection against environmental stresses. In particular, these conidia accumulated substantially higher melanin levels than those produced using the culture medium composition containing a lower sucrose concentration and a higher ammonium phosphate concentration (C:N ratio 10:1) (Figure 4), a characteristic that coincided with the increased thermotolerance observed under these conditions. Conversely, conidia produced using the latter culture medium composition exhibited higher relative ergosterol content. Although ergosterol is essential for membrane organization and integrity, its accumulation did not parallel thermotolerance, suggesting that membrane sterol content alone does not explain the functional differences observed among conidia produced under different culture medium compositions.

Additional mechanisms may also contribute to the observed differences in thermotolerance. Recent studies have shown that conidial heat resistance is strongly influenced by the accumulation of compatible solutes, such as trehalose and mannitol, during conidial maturation [18]. These metabolites stabilize proteins and cellular structures under stress conditions and may help explain differences in thermotolerance among fungal propagules. Whether similar mechanisms operate in *T. sayulitensis* HC1 remains to be determined.

Overall, the results demonstrate that culture medium composition influences different components of conidial quality in a non-uniform manner. The medium composition containing a lower sucrose concentration and a higher ammonium phosphate concentration (C:N ratio 10:1) enhanced germination, whereas the medium composition containing a higher sucrose concentration and a lower ammonium phosphate concentration (C:N ratio 50:1) was associated with greater resistance to thermal stress, higher melanization and the phenotypic traits discussed in Section 4.1. Together with the integrated correlation analysis, these findings support the conclusion that the different components of conidial quality are not necessarily coupled and may respond independently to changes in culture medium composition. For *T. sayulitensis* HC1, the culture medium composition used during conidiation appears to play a central role in determining the balance between rapid germination and resistance to environmental stress.

### 4.3. Implications for the Production of Fungal Inoculants

The results obtained in this study have important implications for the production and quality assessment of fungal inoculants. Although propagule quality is frequently evaluated using parameters such as spore yield, viability or germination, increasing attention has been given to the concept that conidial quality encompasses multiple physiological and functional attributes rather than a single quality indicator [48]. The present results support this concept by demonstrating that germination, thermotolerance and different conidial phenotypic traits did not respond uniformly to culture conditions. Conidia exhibiting higher germination percentages did not necessarily show greater thermotolerance, while culture conditions associated with specific phenotypic traits did not always maximize all functional properties. A similar need to consider production and functional performance jointly was demonstrated for *Beauveria bassiana* in a 22 L packed-bed SSF system, in which substrate selection influenced both conidial production and virulence, highlighting the importance of optimizing functional quality rather than conidial yield alone [49]. These observations indicate that conidial quality should be considered a multidimensional trait integrating morphological, physicochemical, physiological and functional attributes.

The apparent uncoupling between germination and thermotolerance observed in *T. sayulitensis* HC1 has practical implications for process optimization. Production strategies designed to maximize a single characteristic may not necessarily improve overall propagule performance. Instead, culture conditions should be selected according to the intended application of the final product. This application-oriented approach is consistent with studies in *Beauveria bassiana*, in which medium components and culture conditions were specifically optimized to produce conidia with enhanced thermotolerance [50]. For *T. sayulitensis* HC1, when rapid germination is prioritized, the culture medium composition containing a lower sucrose concentration and a higher ammonium phosphate concentration (C:N ratio 10:1) may be considered because it enhanced germination in conidia produced under both SSF and SmF. Conversely, when resistance to thermal stress is prioritized, the culture medium composition containing a higher sucrose concentration and a lower ammonium phosphate concentration (C:N ratio 50:1) may be preferable because conidia produced under both culture systems retained greater relative germination after heat exposure. However, the suitability of these production strategies for storage, transport or application under field conditions must be validated directly.

The present study also highlights the importance of considering culture system and culture medium composition as complementary factors influencing conidial quality. SSF promoted the development of conidia displaying distinct phenotypic characteristics, whereas culture medium composition influenced key functional properties such as germination and thermotolerance. Similar observations have been reported in other fungal systems, where environmental conditions and culture medium composition during conidial formation strongly influenced the stress tolerance and biological performance of the resulting propagules [11]. Likewise, optimization of the culture medium and operational conditions for liquid-state fermentation of *Clonostachys rosea* increased conidial production, maintained high germination and yielded inoculum that reduced *Botrytis cinerea* colonization and sporulation in leaf-disk assays [51]. Together, these results suggest that conidial quality is jointly influenced by the physical culture environment and culture medium composition, which together shape the conidial phenotype and its functional performance.

Overall, the findings indicate that production processes for *T. sayulitensis* HC1 could be tailored to obtain conidia with different functional characteristics depending on the desired application. Rather than seeking a single optimal set of culture conditions, it may be more appropriate to define production strategies according to the specific performance attributes required for the final biological product. For industrial implementation, the proposed strategies should be validated at larger scales by jointly assessing conidial yield and productivity, batch reproducibility, downstream recovery and formulation, storage stability and efficacy under the intended application conditions. This stepwise approach may contribute to the rational design and optimization of fungal inoculants with application-specific quality profiles.

### 4.4. Study Limitations

Although this study provides an integrated characterization of the structural, physicochemical, functional and molecular traits of *Talaromyces sayulitensis* HC1 conidia, several limitations should be acknowledged. The two culture medium compositions evaluated differed in sucrose and ammonium phosphate concentrations and, consequently, in C:N ratio, preventing the independent contribution of these variables from being assessed. In addition, the study focused on end-point conidial characterization and did not include a kinetic evaluation of submerged culture. Therefore, variables such as mycelial biomass accumulation, pH changes, substrate consumption, and conidial production over time were not monitored during SmF. This limits the interpretation of the physiological state of submerged cultures at the time of conidial harvest.

Only a selected set of conidiation-related genes was analyzed, and other regulatory pathways potentially involved in conidial development were not investigated. Therefore, the molecular analyses should be interpreted as complementary evidence supporting the observed phenotypic variation rather than as a comprehensive characterization of the regulatory mechanisms underlying conidial development. Furthermore, the rodlet-like surface structures were examined qualitatively by AFM and SEM, but differences in their abundance and the molecular composition of the putative hydrophobin-containing layer were not quantified. Consequently, the present observations do not establish quantitative differences in the rodlet layer among culture conditions.

Germination and thermotolerance were evaluated using water agar under controlled laboratory conditions. These assays should therefore be considered laboratory-based rapid-screening indicators and cannot be directly equated with conidial survival, establishment or efficacy under field conditions. No greenhouse pot or field experiments were conducted to validate the biological performance of the resulting conidia.

Future studies should independently vary sucrose and ammonium phosphate concentrations to distinguish their individual effects from those of the resulting C:N ratio. In addition, kinetic characterization of submerged fermentation, including biomass accumulation, pH changes, substrate consumption, and conidial production over time, would provide valuable information for physiological interpretation and process optimization. Broader transcriptomic, proteomic and metabolomic analyses will be required to characterize the regulatory pathways and physiological mechanisms associated with the observed phenotypes. Quantitative characterization of rodlet abundance and hydrophobin composition should also be performed using complementary structural and protein-specific approaches. Finally, the candidate production strategies identified here should be evaluated through storage and formulation studies, followed by greenhouse pot experiments and field trials under relevant environmental conditions. Relating laboratory measurements of germination and stress tolerance to persistence, establishment and biological efficacy under these conditions will be necessary to determine the practical value of the conidial quality indicators evaluated in this study.

## 5. Conclusions

The principal contribution of this study is the demonstration, through an integrated analysis of morphological, ultrastructural, physicochemical, functional and selected molecular attributes, that culture system and culture medium composition influence distinct and not necessarily coupled dimensions of conidial quality in *Talaromyces sayulitensis* HC1. Culture system was mainly associated with differences in conidial phenotype, including conidial size and shape, melanization and relative ergosterol content, while outer cell wall thickness was influenced by both culture system and culture medium composition. Culture medium composition differentialy influenced germination and thermotolerance. The contrasting responses of these functional attributes show that conidial quality cannot be adequately represented by a single measurement or maximized through a universally optimal set of culture conditions. This multidimensional and application-oriented interpretation constitutes the main conceptual advance of the study.

The results also support the proposal of two candidate production strategies: the culture medium composition containing a lower sucrose concentration and a higher ammonium phosphate concentration (C:N ratio 10:1) when rapid germination is prioritized, and the composition containing a higher sucrose concentration and a lower ammonium phosphate concentration (C:N ratio 50:1) when greater resistance to thermal stress is required. Future research should determine whether these laboratory-defined quality profiles remain reproducible during process scale-up and translate into stable and effective fungal inoculants under practical application conditions.

## Figures and Tables

**Table 1:** Culture systems and culture medium compositions evaluated for the production of *Talaromyces sayulitensis* HC1 conidia.

Treatment	Culture System	Sucrose (g/L)	(NH_4_)_2_HPO_4_ (g/L)	C:N Ratio
S10-SmF	Submerged	10	0.46	50:1
S10-SSF	Solid	10	0.46	50:1
S5-SmF	Submerged	5	1.09	10:1
S5-SSF	Solid	5	1.09	10:1

**Table 2:** Oligonucleotide primers used for the identification and expression analysis of conidiation-related genes.

Primer	Sequence (5′-3′)	Relevant Characteristics
brlA3F	ACCACACTCGACATTCTT	PCR amplification of a genomic fragment of the *brlA* gene
brlA2R	CTTGGTGTAATGAGCGTT	
qbrlA1F	TGGTGCAACCATATCCTCAA	RT-qPCR amplification of the *brlA* transcript
qbrlA1R	GCATCCACTGAGCCATGTTA	
abaA2F	CGAGTTGATTGCGGAATA	PCR amplification of a genomic fragment of the *abaA* gene
abaA2R	GGAGTTCAGGACATTGTT	
qabaA4F	GCACTAACCCACATGTCGAA	RT-qPCR amplification of the *abaA* transcript
qabaA3R	CAACCACCAATCCCGAGTAT	
fluGD4F	AYTYTACCTTCTTCCCCA	PCR amplification of a genomic fragment of the *fluG* gene
fluGD1R	CTCTTTGTTTTCCCAWCC	
qfluG3F	GAACAATGTGGCCCTGAGTT	RT-qPCR amplification of the *fluG* transcript
qfluG3R	GCAGTGGGAATTTGTCCTTC	
rodA1F	GGTGACCAGGTTGAGGTT	PCR amplification of a genomic fragment of the *rodA* gene
rodA6R	TGGCAGCAAGCAATGTTC	
qrodA1F	CAAAGCAATGTTCTGCTTGC	RT-qPCR amplification of the *rodA* transcript
qrodA1R	CGACAAGTGCTCCAAACTCA	
qact1F	TGTCTTCGAGACCTTCAACG	RT-qPCR amplification of the *act1* transcript
qact1R	ACCGGAGTCAAGGACGATAC	
qtub1F	GCATTGGTACACTGGTGAGG	RT-qPCR amplification of the *tub1* transcript
qtub1R	GATGGAGGCATCCTGGTACT	

Abbreviations: PCR, polymerase chain reaction; RT-qPCR, reverse transcription quantitative polymerase chain reaction. F and R in primer names indicate forward and reverse primers, respectively.

**Table 3:** Morphological, ultrastructural and nanomechanical attributes of *Talaromyces sayulitensis* HC1 conidia produced under different culture conditions.

Treatment	Projected Area (µm^2^)	Circularity Index	Outer Wall Thickness (nm)	Young’s Modulus (MPa)
S10-SSF	2.911 ± 0.367 ^a^	0.927 ± 0.108 ^b^	84.327 ± 36.274 ^b^	329.531 ± 140.235 ^a^
S10-SmF	3.609 ± 0.727 ^b^	0.860 ± 0.102 ^a^	68.212 ± 30.934 ^b^	303.273 ± 184.760 ^a^
S5-SSF	2.877 ± 0.233 ^a^	0.901 ± 0.092 ^a,b^	75.676 ± 31.879 ^b^	222.802 ± 234.692 ^a^
S5-SmF	3.754 ± 0.503 ^b^	0.868 ± 0.101 ^a^	20.955 ± 6.820 ^a^	439.976 ± 211.824 ^a^
Culture system	<0.0001	<0.0001	<0.0001	0.617
Culture medium composition	0.3290	0.4554	0.0001	-
Interaction	0.1195	0.1418	0.0024	-
Model	<0.0001	0.0001	<0.0001	-

Treatments correspond to the combinations of culture system (solid-state fermentation, SSF, or submerged fermentation, SmF) and culture medium composition (S10 or S5). Values are presented as mean ± standard deviation. Different letters within each column indicate significant differences among treatments (*p* < 0.05). The lower rows show two-way ANOVA *p* values. For Young’s modulus, the overall two-way ANOVA model was not statistically significant (*p* = 0.617); therefore, *p* values for the individual factors and their interaction are not presented.

## Data Availability

The data presented in this study are available from the corresponding author upon reasonable request.

## Supplementary Materials

The following supporting information can be downloaded at: https://www.mdpi.com/article/10.3390/jof12080628/s1, Figure S1: UV–Vis absorption spectra of the melanin standard and melanin extracted from *Talaromyces sayulitensis* HC1 conidia; Figure S2: Effect of culture medium composition on conidial production under solid-state and submerged fermentation; Figure S3: Relative expression of conidiation-related genes in response to medium composition.
