# Supplementary material for: Culture Conditions Shape the Phenotype and Quality of Talaromyces sayulitensis HC1 Conidia
Source: J Fungi (Basel). 2026 Aug 21;12(8):628. doi: 10.3390/jof12080628 (PMC13514681; doi:10.3390/jof12080628)
Supplement: Supplementary file 1 [file jof-12-00628-s001.zip › jof-4441987-supplementary.pdf]

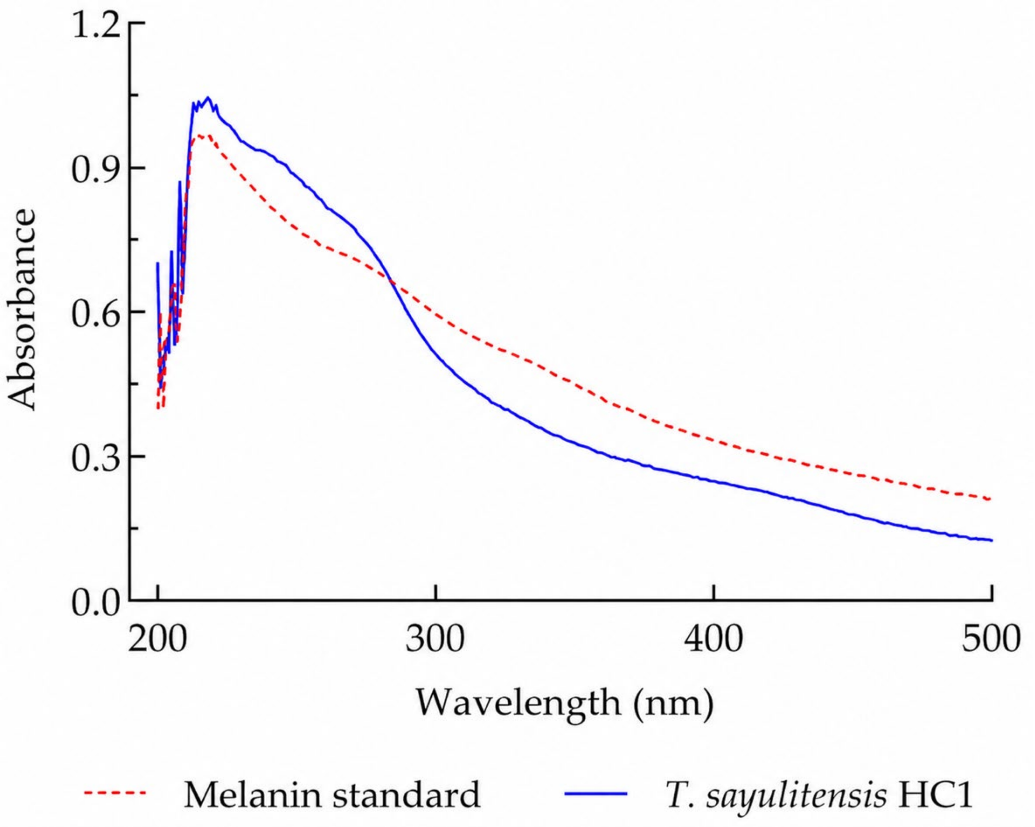

Figure S1. UV–Vis absorption spectra of the melanin standard and melanin extracted from *Talaromyces sayulitensis* HC1 conidia. UV–Vis absorption spectra (200–500 nm) of the melanin standard (Sigma-Aldrich M8631) and melanin extracted from *T. sayulitensis* HC1 conidia. Both samples exhibited a characteristic absorption peak at 218 nm, which was used for melanin quantification.

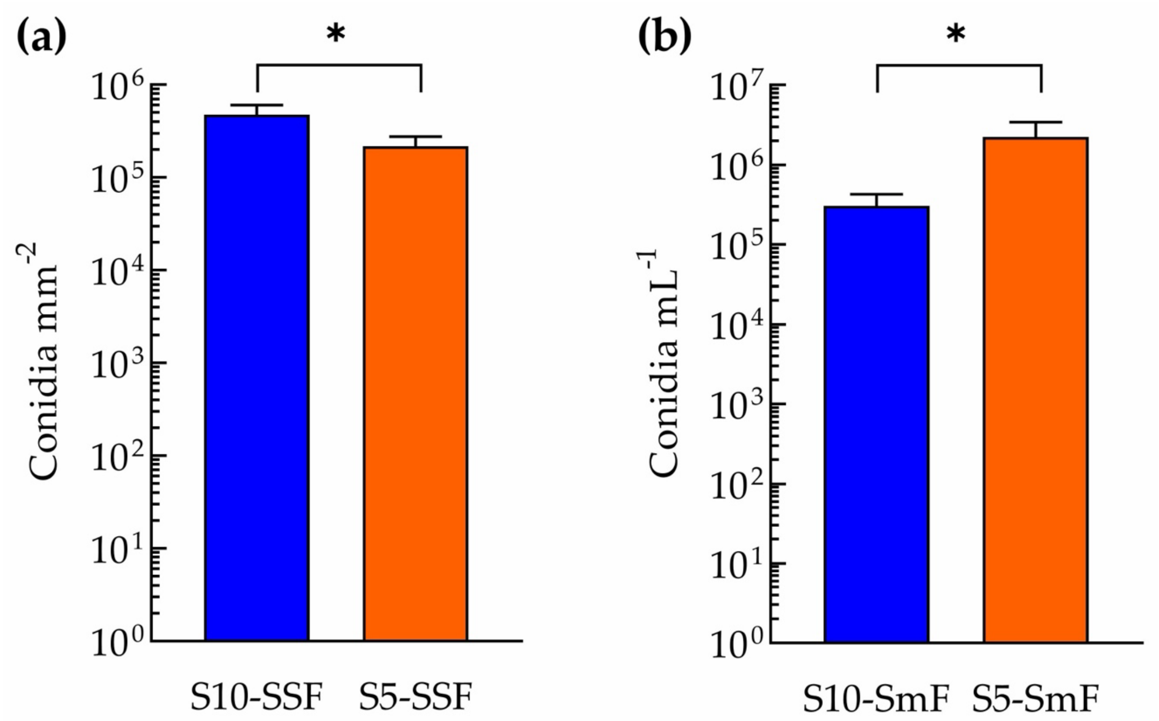

Figure S2. Effect of the nutritional conditions evaluated on conidial production under solid-state and submerged fermentation. (a) Conidial production under solid-state fermentation (SSF). (b) Conidial production under submerged fermentation (SmF). Data are presented as mean ± standard deviation. Asterisks indicate statistically significant differences between the two nutritional conditions within each culture system ($P < 0.05$).

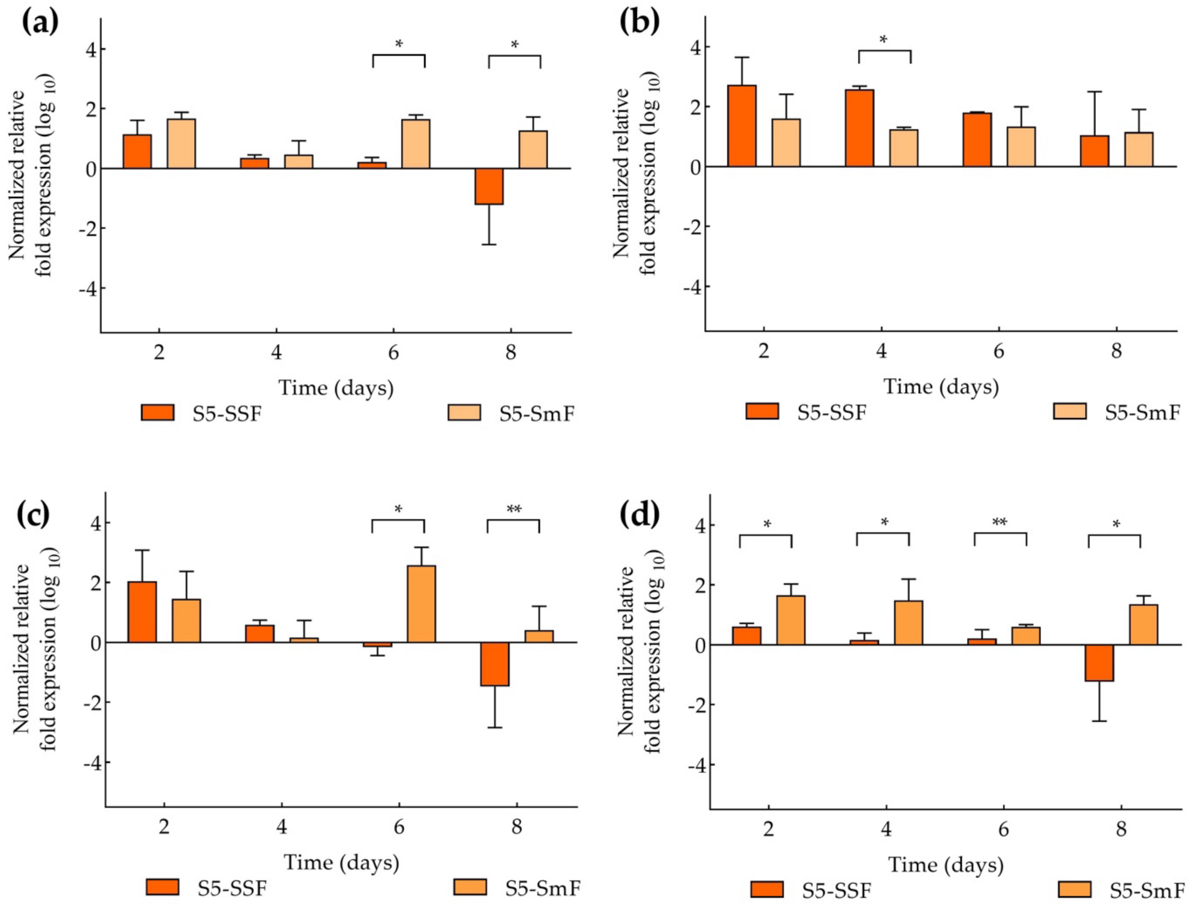

Figure S3. Relative expression of conidiation-related genes in response to the nutritional conditions evaluated. Relative expression profiles of (a) *brlA,* (b) *abaA*, (c) *fluG* and (d) *rodA* in cultures grown under the low-sucrose nutritional condition relative to the corresponding high-sucrose nutritional condition. Expression levels were normalized using *act1* and *tub1* as reference genes and calculated according to the Pfaffl method. Values are expressed as log_{10}-transformed normalized relative quantity (NRQ). Asterisks indicate statistically significant differences between the two nutritional conditions at each sampling time (*P < 0.05; **P < 0.01).
